# Inorganic Nanorods Enable the Memorization of Photoinduced Microlens Arrays in Dye-Doped Liquid Crystals

**DOI:** 10.1021/acsami.4c15591

**Published:** 2024-12-05

**Authors:** Jose Carlos Mejia, Kyohei Hisano, Miho Aizawa, Kohsuke Matsumoto, Takanori Fukushima, Shoichi Kubo, Atsushi Shishido

**Affiliations:** 1 Laboratory for Chemistry and Life Science, Institute of Integrated Research, Institute of Science Tokyo, R1−12, 4259 Nagatsuta, Midori-ku, Yokohama 226-8501, Japan; 2 Department of Chemical Engineering, School of Materials and Chemical Technology, Institute of Science Tokyo, 2-12-1 Ookayama, Meguro-ku, Tokyo 152-8552, Japan; 3 PRESTO, JST, 4-1-8 Honcho, Kawaguchi 332-0012, Japan; 4 Research Center for Autonomous Systems Materialogy (ASMat), Institute of Integrated Research, Institute of Science Tokyo, 4259 Nagatsuta, Midori-ku, Yokohama 226-8501, Japan

**Keywords:** memory effect, microlens array, nonlinear optics, liquid crystals, inorganic nanorods

## Abstract

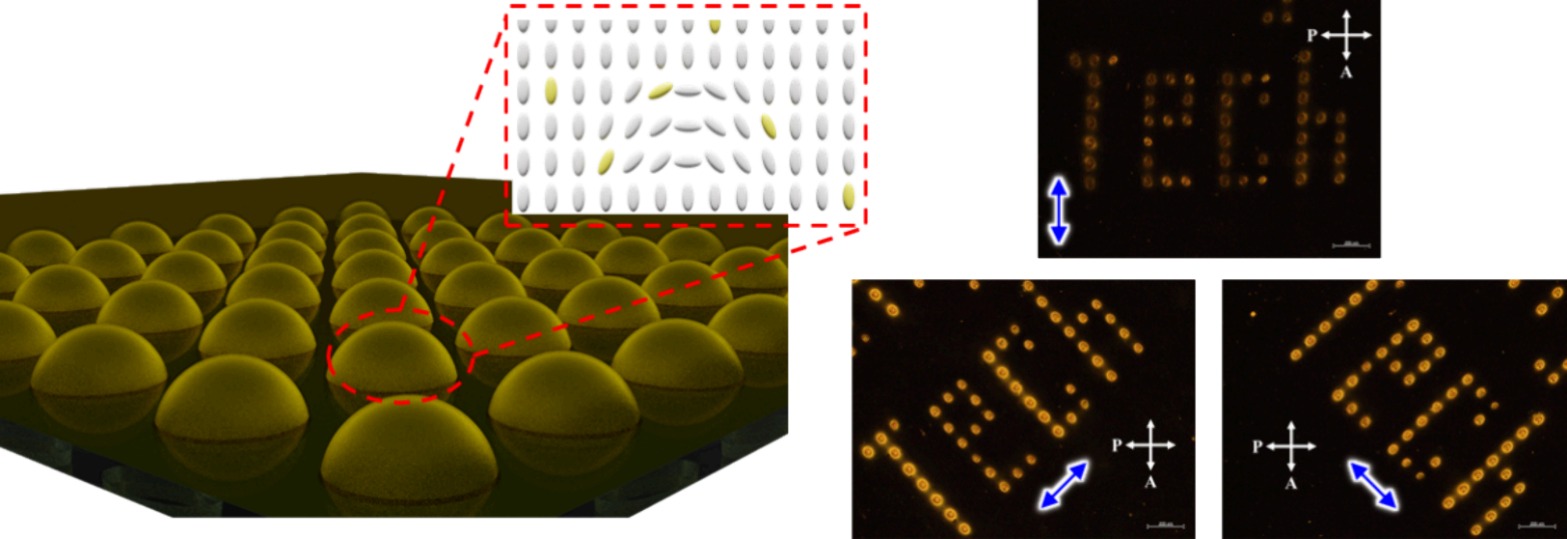

The photoinduced molecular reorientation of liquid crystals (LCs) caused by their nonlinear optical responses has attracted much attention due to their large refractive index change, leading to promising applications in optical devices. This reorientation is typically induced by light irradiation above a threshold intensity and is temporary, with the initial orientation recovering unless the LCs are polymerized and cross-linked. Our report highlights the memory effect of molecular reorientation in LCs. The high-intensity laser beam irradiation of dye-doped LCs containing polymer-grafted ZnO nanorods resulted in the molecular reorientation of the LCs. The effect was maintained even after the light was turned off. This memorized molecular orientation functioned as a polarization-dependent microlens due to the self-phase modulation and self-focusing effect of the propagating light. The polarization selectivity of the microlens allows for innovative optical applications, including security printing and anticounterfeiting.

## Introduction

Microlens arrays are micro-optical systems that contain a collection of miniaturized patterned lenses with diameters less than a few millimeters. They have become increasingly important in various fields, including optical interconnections,^1,2^ imaging sensors,^3^ beam shaping,^4^ optical switches,^5^ light-emitting diodes (LEDs),^6^ photolithography,^7^ miniaturized sensors,^8^ and 3D display systems.^9^ Researchers have shown great interest in fabricating microlens arrays due to their versatility and potential applications. Various methods have been used to fabricate microlens arrays, including lithography,^7,8^ hot compression,^10^ laser writing,^11^ wet etching,^12^ photoresist reflow,^13^ droplet method,^14^ and inkjet printing.^15^ However, not all of these methods are suitable for industrial applications due to their requirement for expensive and sophisticated optical equipment, and some are unable to generate repetitive patterns of microlens arrays. In 2002, Wu et al. introduced a simple method for fabricating microlens arrays using microlens projecting lithography (μLPL) and gray scale masks, which replaced previous techniques.^16,17^ However, the μLPL technique involves photoresist and other challenging lithography techniques. New techniques have been developed to manufacture concave-shaped microlens arrays for applications such as diffusers and compound refractive lenses for focusing X-rays.^18,19^ In recent years, investigations have focused on the fabrication of microlenses using low-cost procedures such as polymeric materials as the substrate. Technological approaches for fabricating a microlens array involve self-developing photopolymers.^20,21^ This is because they do not require any layer development to produce optical microstructures.

Liquid crystals (LCs) are also promising materials for fabricating microlens arrays due to the characteristics of large optical anisotropy and molecular reorientation. LCs can be tailored for switchable and tunable lenses.^22−28^ In a previous report, we presented an innovative method for rapidly producing microlenses by utilizing a combination of photoinduced molecular reorientation of dye-doped LC molecules.^29^ LCs that are doped with dye molecules exhibit a nonlinear optical effect (NLO) when exposed to a polarized light source, which is caused by the molecular reorientation of LCs.^30−42^ We have developed several methods to enhance the optical nonlinearity, including polymer stabilization^43−46^ and polymer-grafted nanorods.^47^ The resulting molecular orientation acts as microlenses due to the induced self-focusing effect. Microlens arrays can be fabricated by fixing the oriented molecules using polymer networks.^29^ The focal length and the radius of curvature, essential parameters for microlenses, can also be tuned by adjusting the light intensity during fabrication.^48^ Additionally, thermal control of surface topography can also be used to adjust the focal length of LC-based microlens arrays.^49^

One possible approach for fabricating microlens arrays based on LCs is to utilize the memory effect of the molecular alignment. The memory effect of LCs has been extensively studied over the past few decades. However, its application to the fabrication of microlens arrays has not yet been reported.^50,51^ This memory effect has been observed under the influence of an external field when shape memory polymers^52^ or nanoparticles^53−55^ are doped into LCs, but it does not show a permanent memory configuration. Nanoparticles in LCs are of significant interest in the microelectronics industry due to their ability to enhance electro-optical properties, memory effect, phase behavior, and switching times.^56−60^ However, issues arise when nanoparticles are not well miscible in the host LC, leading to agglomeration and poor performance in almost all optical applications. Our motivation is to provide an innovative material that can create microlens arrays with the permanent memory effect of LCs and polarization selectivity.

Herein, we present a novel and straightforward approach to fabricate a uniformly patterned microlens array with permanently memorized polarization-dependent LCs with low optical focal length. This is achieved using polymer-grafted zinc oxide (ZnO) nanorods incorporated into dye-doped LCs. The study demonstrated that the molecular reorientation of dye-doped LCs containing polymer-grafted nanorods persisted even after the high-intensity light source was removed. This permanent molecular reorientation resulted in the formation of microlenses that exhibited polarization dependence. The results indicate that this material may be a promising candidate for security and anticounterfeiting applications, given its ability to exhibit permanent polarization selectivity when incorporated with polymer-grafted nanorods.

## Results and Discussion

### Thermodynamic Properties

**Figure 1:**
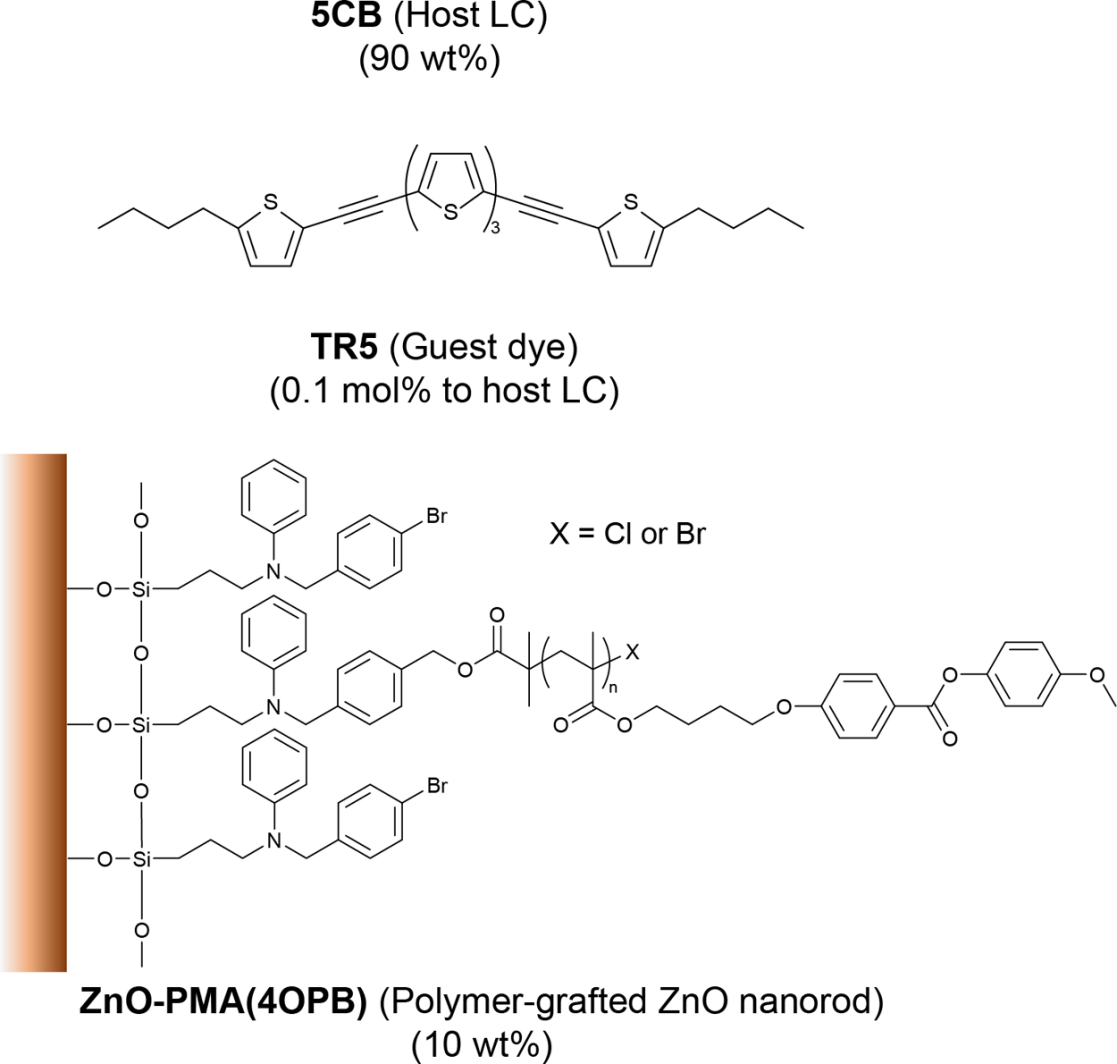
Chemical structures of materials used in this study.

The constituent compounds used in this study are shown in Figure 1 (see also the Materials and Methods section). The composition ratio of 5CB to TR5 was 99.9 to 0.1 mol %, and the weight fraction of the polymer-grafted nanorods dispersed in the TR5-doped 5CB was 10 wt %. Investigating the enhancement in optical nonlinearity when irradiated with a light source requires the polymer-grafted nanorods to be miscible in TR5-doped 5CB. The miscibility of the sample was confirmed by measuring the differential scanning calorimetry (DSC) as shown in Figure S1. The prominent peaks in both the endothermic and exothermic correspond to the nematic-to-isotropic phase transition temperature (*T*_NI_) of 5CB at around 34 °C with an enthalpy of 0.40 kJ/mol. The nematic LC polymer PMA(4OPB) exhibits a *T*_NI_ at 103 °C and is used as the polymer grafted from the nanorod surfaces.^61^ The DSC thermogram of the mixture did not show any peaks derived from PMA(4OPB), indicating that the polymer-grafted nanorods were miscible with TR5-doped 5CB. The small peaks observed during the cooling and heating processes were caused by the presence of nonuniformly stabilized 5CB, which resulted from the slight agglomeration of nanorods. The phase transition behaviors were also investigated by polarized optical microscope observation (see Supporting Information, Figure S2). During the heating process, the images displayed a gradual darkening from 37 °C, reaching complete darkness at 47 °C. This indicates that the phase transition to the isotropic phase was completed. The image remained dark up to 120 °C, which is above the phase transition temperature of the LC polymer grafted from nanorods. Upon cooling, the image exhibited a brightening trend from 40 °C, eventually returning to its initial brightness. The results suggest that the sample exhibits a broad phase transition behavior.

### Optical Properties of Homeotropically Aligned LC Cell

**Figure 2:**
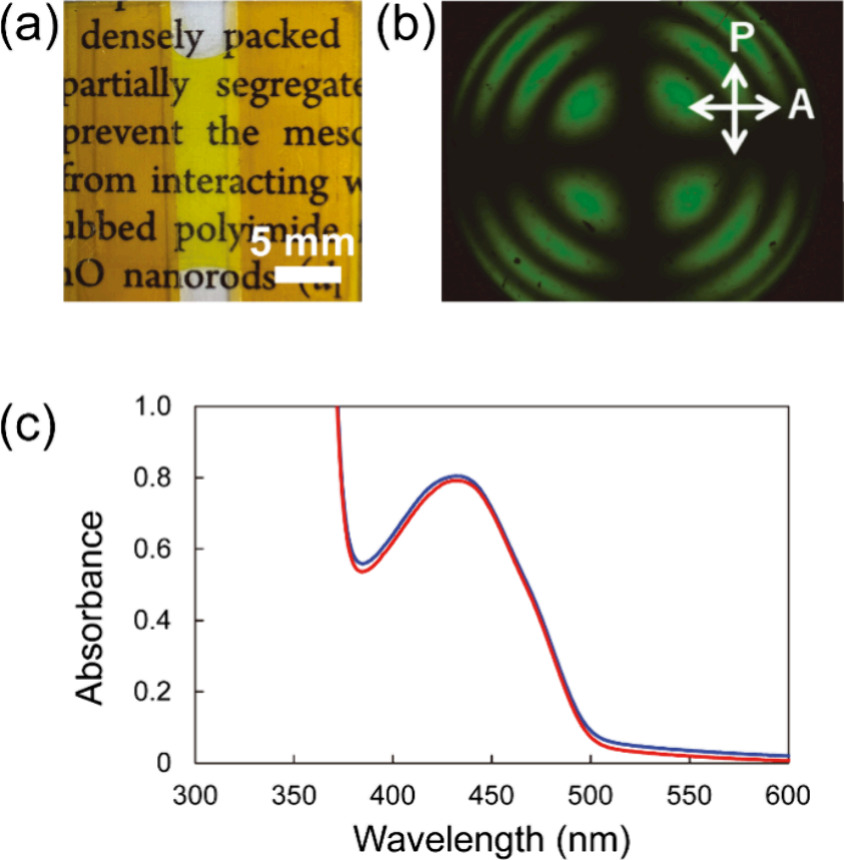
Optical properties of dye-doped LCs containing polymer-grafted nanorods in a 100 μm-thick cell treated with a homeotropic alignment layer: (a) Photograph, (b) conoscopic POM image, and (c) polarized UV–vis absorption spectra parallel (red) and perpendicular (blue) to the sample injection direction.

The mixture was injected into a 100 μm-thick handmade glass cell treated with a homeotropic alignment layer. The obtained LC cell showed a uniform, transparent yellow color over the entire area owing to the TR5 (Figure 2a). The homeotropic alignment of the sample was confirmed by conoscopic polarized optical microscopy (POM) and ultraviolet–visible (UV–vis) absorption spectroscopy. The conoscopic POM image showed a clear isogyre, indicating that molecules were aligned homeotropically (Figure 2b). In addition, the polarized UV–vis absorption spectra parallel and perpendicular to the injected direction of the sample were almost identical in the absorbance range of 5CB and TR5, which also proves the homeotropic alignment of molecules (Figure 2c). The surface modification of nanorods with LC polymers has an important role to obtain uniform mixtures. Addition of bare nanorods without LC polymers in the surface caused significant aggregation, resulting in opaque appearance (see Supporting Information, Text S2 and Figure S3).

### Nonlinear Optical Response of TR5-Doped LCs Containing Polymer-Grafted Nanorods

**Figure 3:**
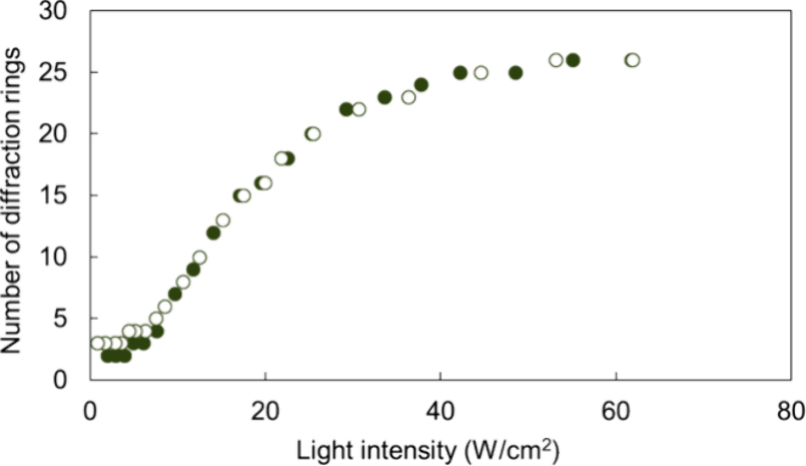
Number of diffraction rings as a function of light intensity for TR5-doped 5CB containing 10 wt % of polymer-grafted ZnO nanorods. Closed and open circles show the results of first and second measurements, respectively.

**Figure 4:**
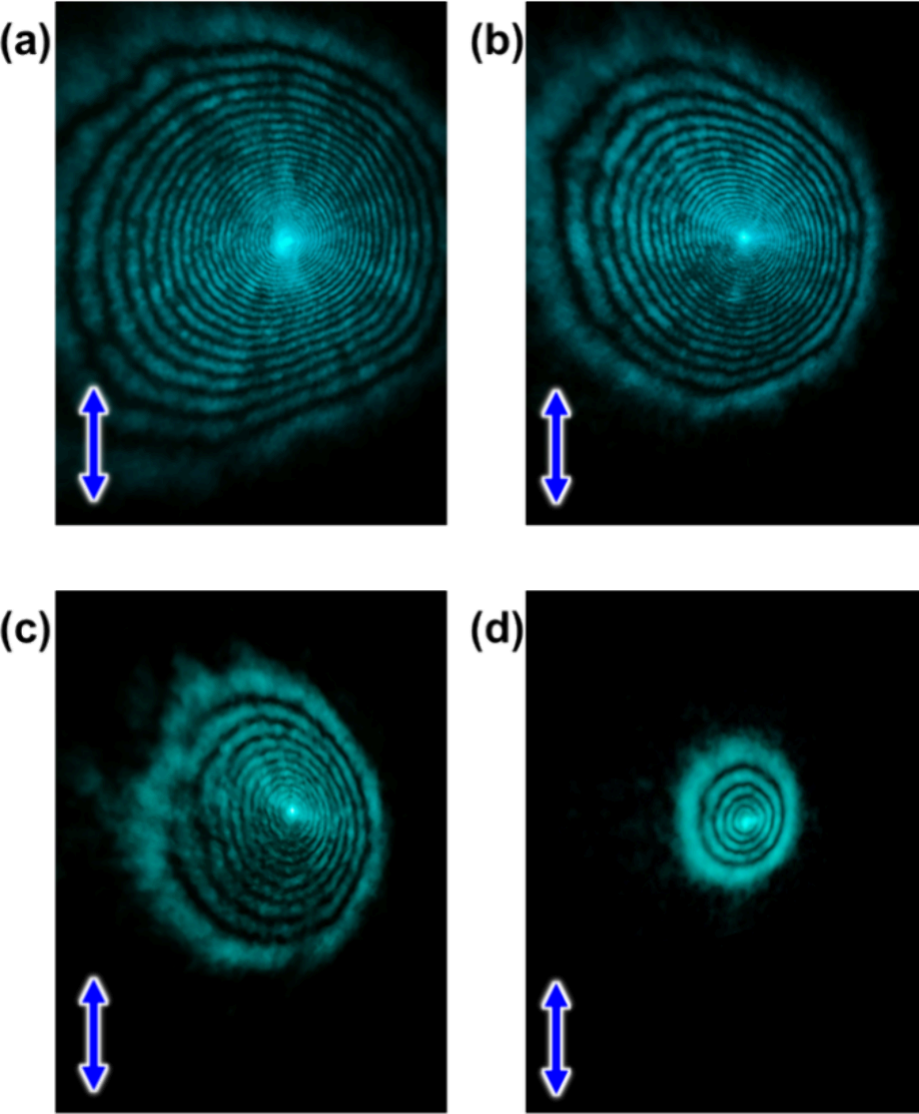
Diffraction ring images of TR5-doped 5CB containing polymer-grafted nanorods with the incident light intensity of (a) 61, (b) 38, (c) 22, and (d) 15 W/cm^2^. Blue arrows indicate the polarization direction of the incident laser beam.

The diffraction rings were analyzed to evaluate the photoinduced molecular reorientation of 5CB when a polarized laser beam was irradiated through the sample. The sample underwent irradiation starting at a light intensity of 65 W/cm^2^, followed by a decrease in light intensity. The number of rings was counted at each light intensity (Figure 3). Photographs of the diffraction rings at each intensity were also taken and are shown in Figure 4. The number of diffraction rings (*N*) is associated with the photoinduced refractive index change (Δ*n*′), which reflects the degree of molecular reorientation. Δ*n*′ is calculated as the total retardation divided by the cell thickness. The irradiation with a Gaussian laser beam induces the molecular orientation distribution, causing the gradual change in the retardation. The total retardation is the integration of the retardation at each depth along the depth direction. This molecular reorientation can be quantitatively characterized by the following expression: Δ*n*′ = *N*λ*L*^–1^, where λ is the wavelength of the irradiated laser beam (488 nm), and *L* is the cell thickness (100 μm).^62^ The degree of molecular reorientation of 5CB increased with light intensity.

### Memory Effect and Microlens Formation

The diffraction rings are expected to cease after the irradiated light source is removed from the sample because the molecules return to their original orientation. In our recent report,^47^ we found that the lowest threshold intensity to induce a single self-diffraction ring with a sample containing 5 wt % of polymer-grafted nanorods in TR5-doped 5CB was 12 W/cm^2^. However, as shown in Figure 3, the diffraction rings did not disappear even at the lowest light intensities in the experiment, 0.8 W/cm^2^. The results indicate that the molecules, including the polymer-grafted nanorods, underwent a permanent molecular reorientation, inducing a lens effect even at low light intensities. Previous research has shown that diffraction rings in polymer-stabilized LC (PSLC) systems can be memorized through photopolymerization in the irradiated area, which homogeneously immobilizes LC molecules.^29^ In contrast, the system studied here demonstrated the memorization of diffraction rings in the irradiated area without photopolymerization.

**Figure 5:**
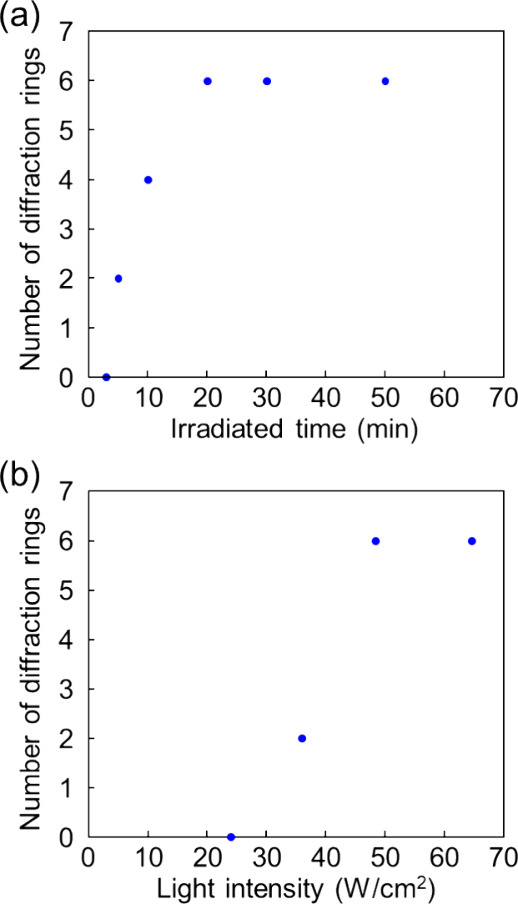
Number of diffraction rings observed using a laser beam at 0.1 W/cm^2^ after the irradiation at different irradiated times with a light intensity of 61 W/cm^2^ (a) and at various light intensities for 30 min (b) followed by 30 min in the dark.

The requirements to store the molecular orientation were investigated by controlling two parameters: the irradiated time with the constant light intensity and the light intensity with the constant irradiated time. The light intensity was set at a constant 61 W/cm^2^, which corresponded to the saturation point for the number of diffraction rings as illustrated in Figure 3, and irradiated for various times. After the elapsed time, the pump laser beam was turned off, and the sample was left in the dark for 30 min. The diffraction rings were observed after irradiating the sample with a low light intensity laser beam of 0.1 W/cm^2^ (Figure 5a). When the sample was irradiated at 61 W/cm^2^ for 50 min, six diffraction rings were observed at 0.1 W/cm^2^. The number of memorized diffraction rings remained unchanged after 20 min of irradiation, but no memorization of diffraction rings occurred after 3 min of irradiation. Subsequently, an investigation was conducted into the effect of light intensity on the memory effect. Figure 5b illustrates the effect of light intensity on the diffraction rings. The diffraction rings were measured using a 0.1 W/cm^2^ laser beam after the irradiation at a certain intensity for 30 min, which was longer than the minimum time to allow for more leeway, followed by 30 min in the dark. Six diffraction rings were observed when the sample was irradiated at 48.5 W/cm^2^, and the number of rings decreased as the irradiation intensity was lowered. The results indicate that a minimum of 61 W/cm^2^ for 20 min or 48.5 W/cm^2^ for 30 min is required to exhibit a memory effect with a high refractive index change.

**Figure 6:**
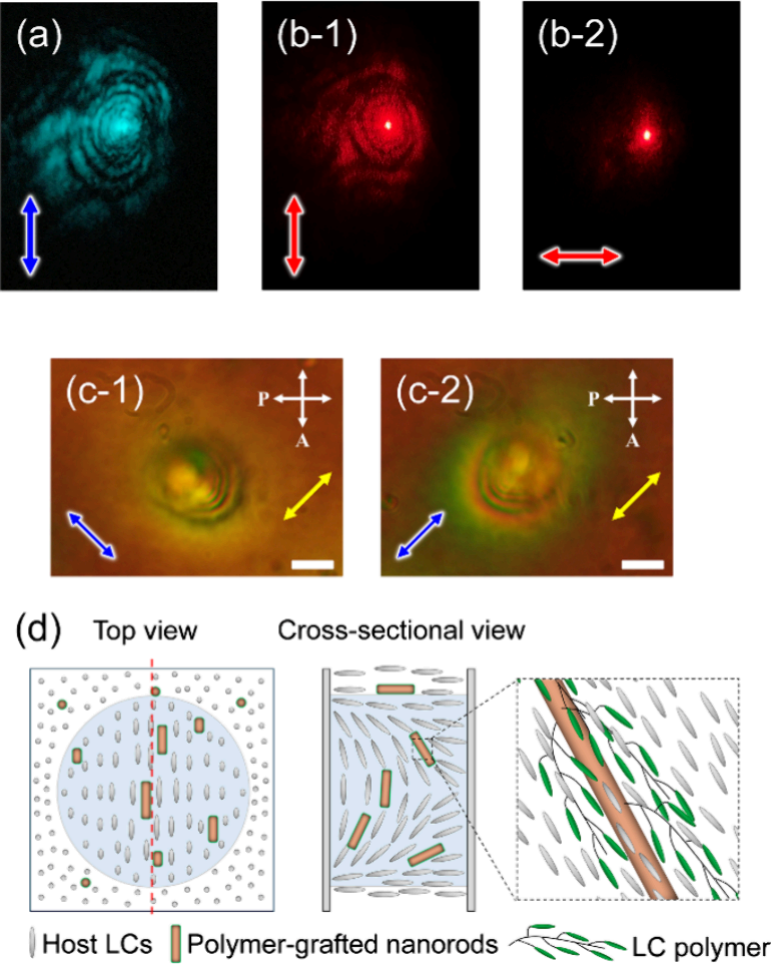
Formation of microlenses by the irradiation with a polarized blue laser beam at 67.5 W/cm^2^ for 30 min. (a) The memorized diffraction rings observed with a polarized blue laser beam at 0.1 W/cm^2^. (b) The memorized diffraction rings probed with a red laser beam polarized parallel (b-1) and perpendicular (b-2) to the polarization direction of the blue laser beam used for molecular reorientation. (c) POM images of the formed microlenses exhibiting memorized diffraction rings by placing the optical axis of a tint plate (retardation, 530 nm) perpendicular (c-1) and parallel (c-2) to the polarization direction of the blue laser beam. The red arrow represents the polarization direction of the probe red laser beam. The yellow arrow represents the direction of the optical axis of the tint plate. The scale bar is 20 μm. (d) Schematic representation of the orientation of TR5-doped 5CB host and polymer-grafted nanorods inside the microlens: top view, cross-sectional view corresponding to the red dotted line on the top view, and magnified illustration. Light blue areas represent the area irradiated with a laser beam to fabricate the microlens.

The formation of microlens with memorized molecular orientation was investigated using a linearly polarized red laser beam with a wavelength of 633 nm as a probe. At this wavelength, the sample does not absorb light. A slightly higher intensity (61 W/cm^2^) and longer time (30 min) than the minimum requirement was employed in order to ensure the stability of the memorized molecular orientation. Figure 6a displays the diffraction ring that was observed with a 0.1 W/cm^2^ blue laser beam passing the fabricated microlens. Red diffraction rings were observed when a red laser beam was directed at the same location with a polarization direction parallel to that of the blue laser beam (Figure 6b-1). By rotating the polarization direction of the red laser beam orthogonally, the red diffraction rings disappeared (Figure 6b-2). This suggests that the molecular alignment of 5CB along the polarization direction of the blue laser beam was induced due to order-to-order molecular reorientation and was memorized.

POM observation confirmed that the molecular orientation causes memorized diffraction rings (Figure 6c). Interference patterns were observed due to the spatial change in the refractive indices of 5CB, induced by nonlinear molecular reorientation. The investigation of the microlens’ polarization dependence was conducted with a tint plate. Subtractive and additive colors were observed when the polarization direction of the microlens was rotated parallel and perpendicular to the optical axis of the tint plate (Figure 6c-1 and 6c-2). This indicates that the long axis of the 5CB molecules located around the microlens had a uniform unidirectional alignment. The 5CB molecules in the center were aligned in a Gaussian-shaped distribution, similar to the shape of the polarized laser beam used to create the microlens, with polarization dependence. Figure 6d shows a schematic representation of the orientation of TR5-doped 5CB and polymer-grafted nanorods inside the microlens. The irradiation with a laser beam induces the orientation distribution of TR5-doped 5CB by the nonlinear effect, and LC polymer-grafted nanorods are cooperatively aligned along 5CB. Once the 5CB and LC polymer-grafted nanorods are oriented, the LC polymer on the nanorods stabilizes the molecular orientation of 5CB and causes the memorization.

It is noteworthy that the memory effect is exhibited only when polymer-grafted nanorods are incorporated. For example, TR5-doped 5CB was unable to memorize the molecular reorientation of 5CB without the inclusion of polymer-grafted nanorods. Additionally, the homopolymer PMA(4OPB) was incorporated into TR5-doped 5CB with the same molar ratio as the polymer grafted on the nanorod, but no memory effect was observed (Figure S5). The system was able to generate polarization-dependent microlenses due to the order-to-order molecular orientation of 5CB, even without the polymer network required in previous works, by incorporating polymer-grafted nanorods.

### Characterization of Microlens Arrays

**Figure 7:**
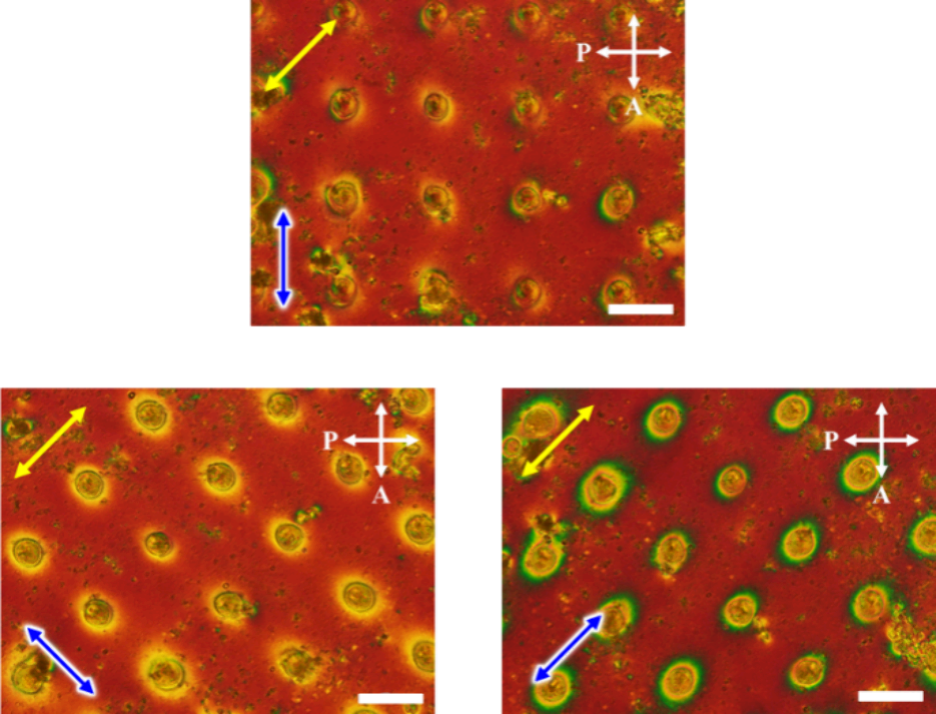
POM images of the spatial distribution of microlenses across the LC cell observed with a tint plate. The stage was rotated to 45° sideways to confirm the polarization dependence of the microlenses. The blue arrow represents the polarization direction of the polarized blue laser beam (light intensity of 67.5 W/cm^2^ for 30 min) used to fabricate each microlens. The yellow arrow represents the direction of the 530 nm tint plate. The scale bar is 100 μm.

A two-dimensional microlens array was fabricated by irradiating a laser beam at different locations to achieve a spatial distribution of microlenses. Figure 7 shows the POM images of the fabricated microlens array. The polarization direction of the microlens array was investigated by using a tint plate, similar to the single microlens above. It was confirmed that there was a positional ordering of 5CB molecules in each microlens, even in the microlens array.

**Figure 8:**
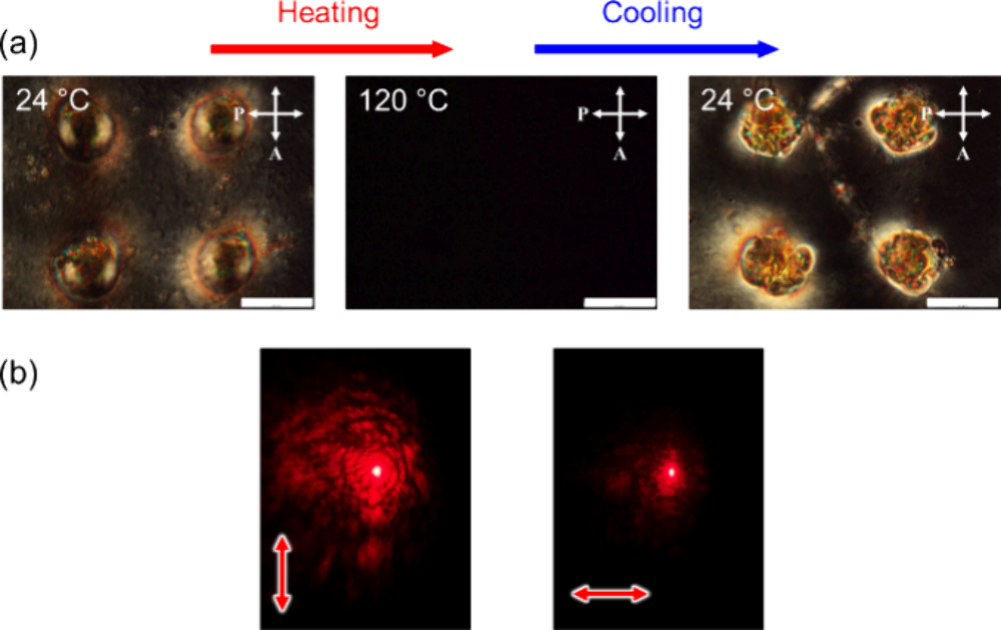
(a) POM images of the microlenses at 24 °C, after heated to 120 °C, and after subsequent cooled to 24 °C. The scale bar is 50 μm. (b) Photographs of probe beam showing polarization dependence. The red arrows represent the polarization direction of the incident probe beam.

The thermal stability of the microlens array was assessed by placing the sample under POM equipped with a heating stage and recording micrographs (Figure 8a). At 120 °C, the image became dark due to the nematic to isotropic phase transition of 5CB and the grafted polymer, PMA(4OPB). The sample was subsequently cooled to room temperature, and the microlens array was recovered. The polymer-grafted nanorods stabilize the molecular orientation of 5CB, and the microlens array remains permanent even at high temperatures. The polarization direction of 5CB molecules in the microlens after annealing was confirmed by probing a red laser beam (Figure 8b), indicating that the molecules return to their original molecular alignment after cooling down the sample in the isotropic phase.

**Figure 9:**
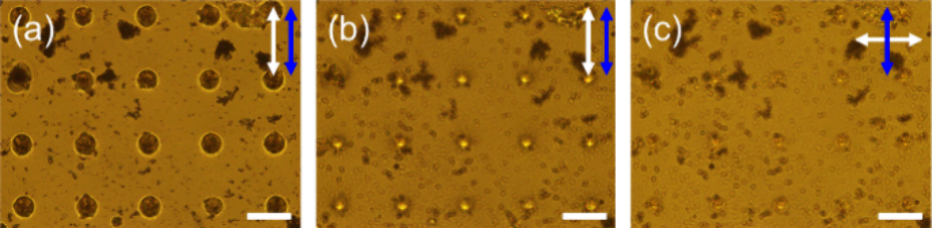
POM images of the microlens array at the base focus (a) and sharp focus point (b,c). POM observation was carried out without an analyzer. The white arrows represent the polarization direction of illumination light of the microscope. The blue arrows indicate the polarization direction of the incident laser beam for fabricating the microlens array. The scale bar is 100 μm.

The investigation was performed on the most prominent feature of a lens, the focal length. The focal length of the microlens array was measured using an optical microscope, as depicted in Figure S6. Initially, the base of the microlens array was focused at *Z* = 0 μm (Figure 9a and Figure S6a). The stage was adjusted until a sharp, focused light was observed on each microlens with incident light linearly polarized parallel to the polarization direction of the microlens (Figure 9b). The distance moved for focusing (Δ*Z*) was defined as the focal length (Figure S6b). The focal length of the microlens array was determined to be 107 ± 4 μm. When the linearly polarized light was set perpendicular to the polarization direction of the microlens, the focused light nearly disappeared (Figure 9c). This demonstrates the polarization selectivity of the microlens array, which arises from the molecular orientation parallel to the polarization direction of the laser beam used during microlens fabrication. We compared the experimental focal length of the microlens array to theoretical values to confirm its reliability. The refractive index induced in the sample was assumed to be semispherical to describe the focal length of the microlens array using the following equation:^63^
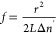
where *r* is the microlens radius, *L* is the cell thickness, and Δ*n*′ is the photoinduced refractive index change of the microlens. The value of Δ*n*′ is calculated as the total retardation divided by the cell thickness and associated with the number of diffraction rings *N* by the equation Δ*n*′ = *N*λ*L*^–1^. In this case, *N* is equal to 6, which corresponds to the number of memorized diffraction rings (Figure 5b). The wavelength of the laser beam used to fabricate the microlens array is λ = 488 nm, and the thickness of the LC cell is *L* = 100 μm. Based on these values, the estimated Δ*n*′ of the fabricated microlens array is 0.0293. The radius of each microlens is approximately 25 μm, as observed in POM images. Theoretical calculation resulted in a focal length of 107 μm, which is almost identical to the experimental value.

### Microlens Arrays with Polarization Selectivity

**Figure 10:**
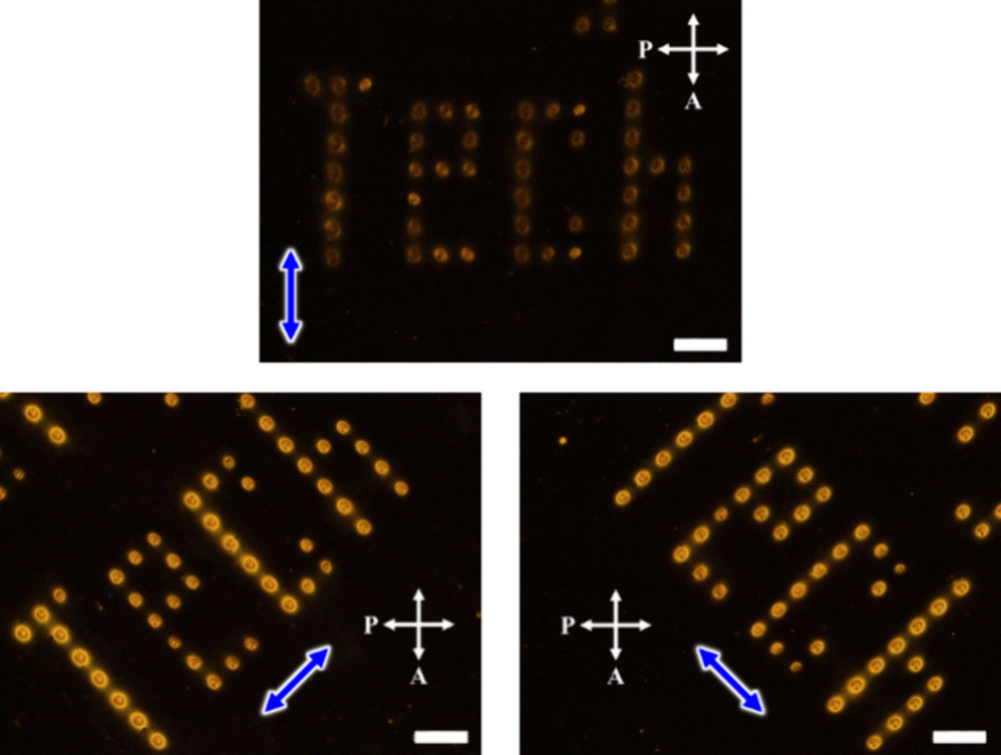
POM micrographs of a microlens array pattern showing the word “Tech.” The sample was rotated 45° sideways to confirm the polarization direction of the molecules in each microlens. Each microlens was fabricated with a light intensity of 90 W/cm^2^ for 10 min. The blue arrows represent the polarization direction of the polarized laser beam used to fabricate each microlens. The scale bar is 200 μm.

Microlenses with small focal lengths that are polarization-dependent have been utilized in optical applications, including security printing and anticounterfeiting. In this study, we investigated the preliminary performance of our system for these applications. The microlenses were spatially distributed using a stage controller to control the *x* and *y*-directions. Figure 10 displays the word “Tech” with polarization dependency printed by irradiating a sample with a laser intensity of 90 W/cm^2^ for 10 min per microlens. The polarization of the microlenses was confirmed by POM. The sample became darker when the polarization was between crossed Nicols and brighter when rotated sideways. The slightly bright state observed at the polarization direction parallel to crossed polarizers is due to some disorder in molecular orientation and light scattering causing depolarization.

**Figure 11:**
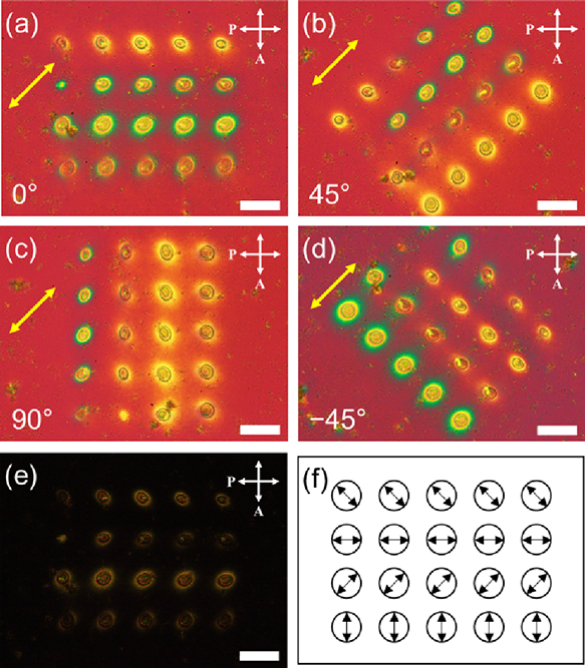
POM images of the microlens array fabricated with different polarization directions. The LC cell containing the microlens array was rotated counterclockwise (a) 0°, (b) 45°, (c) 90°, and (d) –45° equipped with a 530 nm tint plate. (e) POM image of the microlens array with a tint plate. (f) Schematic representation of the molecular alignment in each microlens. Each microlens was fabricated with a light intensity of 90 W/cm^2^ for 10 min. The yellow arrow represents the optical axis of the tint plate. The scale bar is 100 μm.

The direction of polarization for each microlens can be controlled by adjusting the polarization of the laser beam using a half-wave quarter plate, as demonstrated in Figure 11. A 4 × 5 array of microlens was produced with varying polarization directions in each row. The polarization direction was verified using a POM equipped with a tint plate by rotating the sample 45° counterclockwise (Figure 11a-d). Figure 11e displays the POM image of the microlens array without a retarder plate. Based on the interference color chart, the polarization direction was determined by observing the additive and subtractive colors around the microlens as green and yellow, respectively. The polarization direction of the microlenses is schematically represented in Figure 11f. It is noteworthy that the microlens arrays have long-term stability. The arrays could be observed with polarization dependence two years after fabrication, although some aggregation of the nanorods appeared. Furthermore, the stored array enabled the diffraction ring formation by the irradiation of a laser beam (see Supporting Information, Text S6 and Figures S7, S8, and S9). The polarization-dependent microlens array with long-term stability has potential applications in anticounterfeiting materials for high-security level and optical encryption.

## Conclusions

In summary, we have presented an innovative method for producing a polarization-dependent microlens array by incorporating polymer-grafted nanorods into dye-doped LC systems. The molecular reorientation of LCs, which is observed by the propagation of irradiated light forming concentric diffraction rings, is memorized by light irradiation with high intensities. Microlenses can be fabricated since the LC molecules with the resultant molecular orientation exhibit a self-focusing effect. The molecular orientation direction in the microlens was confirmed to be parallel to that of the blue laser beam used for fabricating the microlens through POM observation with a tint plate and the self-diffraction ring formation using a red probe laser beam. The focal length was observed when polarized light parallel to the polarization direction of the LC molecules in the microlens was incident, but disappeared when the incident light polarization was perpendicular. The polarization-dependent microlenses obtained in this study are a promising candidate for a wide range of applications, including security printing and anticounterfeiting, due to their controllable polarization direction.

## Materials and Methods

### Materials

A host LC, 5CB (4-cyano-4′-pentylbiphenyl), was obtained from Merck Ltd., Tokyo, Japan. The guest dye, TR5 (5,5″-bis(5-butyl-2-thienylethynyl)-2,2′:5′,2″-terthiophene), was synthesized as previously reported.^64^ The polymer-grafted nanorods, ZnO-PMA(4OPB) (poly(4-[4-(4-methoxyphenyloxycarbonyl)phenoxy]butyl methacrylate)), were synthesized as previously reported.^61^ Tetrahydrofuran (THF with stabilizer, 99.5%) and 2-propanol (IPA, 99.5%) were purchased from Fujifilm Wako Pure Chemical Corp., Osaka, Japan.

### Sample Preparation

A solution of 0.01 M TR5 was added to 5CB, stirred for 1 h, and dried under vacuum overnight. The TR5 concentration to 5CB concentration was 0.1 mol %. The polymer-grafted nanorods were dispersed in THF by sonication and added to the TR5-doped 5CB mixture. The solvent was evaporated under vacuum overnight. The composition ratio between the TR5-doped 5CB to ZnO-PMA(4OPB) was 90 to 10 wt %.

### LC Cell Preparation

Two commercially available glass substrates (2.5 cm × 2.5 cm) were ultrasonically cleaned with IPA for 30 min and subsequently treated with a UV-ozone cleaner (NL-UV42, Nippon Laser & Electronics Lab. Co. Ltd., Nagoya, Japan) for 10 min. The cleaned glass substrates were immersed in a 200 mL ethanol solution containing 0.4 g of a silane coupling agent (octadecyltrimethoxysilane, Tokyo Chemical Industry Co., Ltd., Tokyo, Japan) for 30 min and annealed at 120 °C for 2 h to yield glass substrates modified with a silane coupler. The LC cell was handmade by sandwiching two surface-treated glass substrates with 100 μm-thick polyimide tapes. The sample was injected into the fabricated LC cell by capillary forces at the isotropic phase of 5CB at 70 °C and gradually cooled to room temperature at 2 °C/min. The LC cell was stored overnight to stabilize the molecular reorientation of 5CB, TR5, and polymer-grafted nanorods.

### Characterization

The thermodynamic property of the sample was measured using a differential scanning calorimeter (DSC, Hitachi High-Tech Corp., DSC7000X, Tokyo, Japan) at a cooling and heating rate of 1 °C/min. The initial molecular alignment of the sample after injection into the LC cell was confirmed with a conoscopic polarized optical microscope (POM, BX50, Olympus, Tokyo, Japan) equipped with an interference filter at 545 nm. The polarized absorption spectrum of the sample was measured with an ultraviolet–visible (UV–vis) absorption spectrophotometer (V-670, JASCO Corp., Tokyo, Japan) equipped with a rotatable polarizer to confirm the TR5 orientation.

### Evaluation of Nonlinear Molecular Reorientation and Formation of the Microlens Array

The optical setup used to induce photoinduced molecular reorientation and form microlenses is shown in Figure S4a. A linearly polarized blue laser beam with a wavelength of 488 nm (EXLSR-488C-200-CDRH, Spectra-Physics, MKS Instruments, Inc., Milpitas, California, USA) was incident to the LC cell. The light intensity was controlled using a variable neutral density filter. The incident laser beam power *W*_0_ on the LC cell was calculated by taking the ratio of the beam power split by a nonpolarizing cube beam splitter. The light intensity at the irradiation spot was defined as *I* = *W*_0_/πr^2^, where *r* is the radius of the laser beam at the focal point of L4, which was controlled to be 50 μm. The laser beam diameter at the focal point was 50 μm. The LC cell was exposed to the laser beam with sufficiently high intensity giving rise to the formation of concentric diffraction ring patterns observed on a white screen placed behind the LC cell. Then, the light intensity was gradually decreased while visually counting the number of rings. Microlenses were obtained by irradiating the sample at high light intensities for a prolonged time. The microlens array was fabricated by irradiating a high-intensity laser beam for 30 min each spot at different locations to form a spatial distribution of microlenses by placing the sample in an *x-y* biaxial stage. A half waveplate (WPQ-4880–2M, Sigma Koki Co. Ltd., Tokyo, Japan) was inserted before L4 to change the polarization direction of the incident laser beam as necessary. The molecular orientation in fabricated microlenses was probed with a 633 nm polarized red laser beam (Melles Griot, 05 LHP 151, Pneum, Saitama, Japan) (Figure S4b). The polarization direction of the probe beam was adjusted to be parallel and perpendicular to the polarization direction that caused the formation of the microlenses. The polarization direction of 5CB in the microlens was further investigated with an orthoscopic POM equipped with a 530 nm retarder plate (U-TP530, Olympus, Tokyo, Japan). The thermal characteristics of the microlens array were investigated with orthoscopic POM equipped with a temperature controller stage (HCS302-MK1000, INSTEC, Inc., Boulder, Colorado, USA).

### Focal Length Measurement

The focal length of the microlens array was experimentally measured under POM by removing the analyzer and sending a linearly polarized light parallel to the polarization direction of the LC molecules in the microlens, as shown in the schematic in Figure S6. The base of the microlens was first focused, and this distance was defined as *Z* = 0 μm. The stage height was then adjusted until a sharp light point was observed on top of the microlens array, and the distance moved was defined as the focal length. This measurement was performed six times to estimate the average focal length.

## Supporting Information Available

LC properties of TR5-doped 5CB containing polymer-grafted ZnO nanorods; miscibility of bare ZnO nanorods with TR5-doped 5CB; optical setup to fabricate and evaluate microlenses; formation of diffraction rings through TR5-doped 5CB containing homopolymer; measurement of the focal length of the microlens; long-term stability of microlens arrays (PDF)
