# Supplementary material for: Inorganic Nanorods Enable the Memorization of Photoinduced Microlens Arrays in Dye-Doped Liquid Crystals
Source: ACS Appl Mater Interfaces. 2024 Dec 5;16(50):69881–90. doi: 10.1021/acsami.4c15591 (PMC11660039; doi:10.1021/acsami.4c15591)
Supplement: Supplementary file 1 — am4c15591_si_001.pdf [file am4c15591_si_001.pdf]

Supporting Information

Inorganic Nanorods Enable the Memorization of Photoinduced Microlens Arrays in Dye-Doped Liquid Crystals

Jose Carlos Mejia[1,2], *Kyohei Hisano*[1,2], *Miho Aizawa*[1,2,3], *Kohsuke Matsumoto*[1,2], *Takanori Fukushima*[1,2,4], *Shoichi Kubo*[1,2,*], *Atsushi Shishido*[1,2,4,*]

[1] Laboratory for Chemistry and Life Science, Institute of Integrated Research, Institute of Science Tokyo, R1–12, 4259 Nagatsuta, Midori-ku, Yokohama 226-8501, Japan

[2] Department of Chemical Engineering, School of Materials and Chemical Technology, Institute of Science Tokyo, 2-12-1 Ookayama, Meguro-ku, Tokyo 152-8552, Japan

[3] PRESTO, JST, 4-1-8 Honcho, Kawaguchi 332-0012, Japan

[4] Research Center for Autonomous Systems Materialogy (ASMat), Institute of Integrated Research, Institute of Science Tokyo, 4259 Nagatsuta, Midori-ku, Yokohama 226-8501, Japan

*E-mail: kubo@res.titech.ac.jp (Shoichi Kubo), ashishid@res.titech.ac.jp (Atsushi Shishido)

This file includes:

Text S1. Liquid-crystalline (LC) properties of TR5-doped 5CB containing polymer-grafted ZnO nanorods.

Text S2. Miscibility of bare ZnO nanorods with TR5-doped 5CB.

Text S3. Optical setup to fabricate and evaluate microlenses.

Text S4. Formation of diffraction rings through TR5-doped 5CB containing homopolymer.

Text S5. Measurement of the focal length of the microlens.

Text S6. Long-term stability of microlens arrays.

Figure S1. DSC thermogram of dye-doped LCs containing polymer-grafted nanorods.

Figure S2. POM images of TR5-doped 5CB

Figure S3. Photograph and POM image of TR5-doped 5CB containing bare nanorods, and photograph of transmitted light.

Figure S4. Optical setup used to fabricate and evaluate the microlens.

Figure S5. Photographs of the diffraction rings of TR5-doped 5CB containing homopolymer.

Figure S6. Schematic representation for measuring the focal length of microlenses.

Figure S7. POM images of microlens array pattern showing the word "Tech" observed two years after fabrication.

Figure S8. POM images of the microlens array with different polarization directions observed two years after fabrication.

Figure S9. Photograph of diffraction rings through the microlens two years after fabrication.

Text 1. Liquid-crystalline (LC) properties of TR5-doped 5CB containing polymer-grafted ZnO nanorods.

The DSC data during the heating process exhibited an endothermic peak at approximately 34 °C, which is attributed to the nematic-to-isotropic phase transition of 5CB (Figure S1). According to POM observation (Figure S2), the images displayed a gradual darkening from 37 °C, reaching complete darkness at 47 °C during the heating process. This suggests that the TR5-doped 5CB containing polymer-grafted nanorods becomes isotropic within the temperature range of 34–47 °C. The image remained dark up to 120 °C, which is above the phase transition temperature of the LC polymer grafted from nanorods. Upon cooling, the image exhibited a brightening trend from 40 °C, eventually returning to its initial brightness.

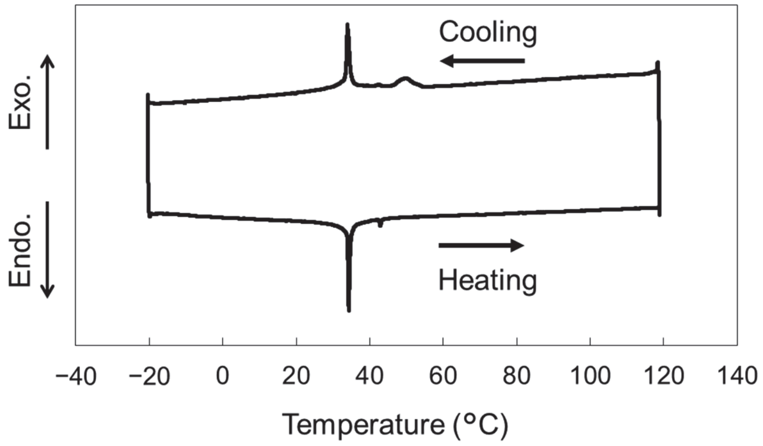

Figure S1. DSC thermogram of TR5-doped 5CB containing polymer-grafted nanorods. Scanning rate: 1 °C/min.

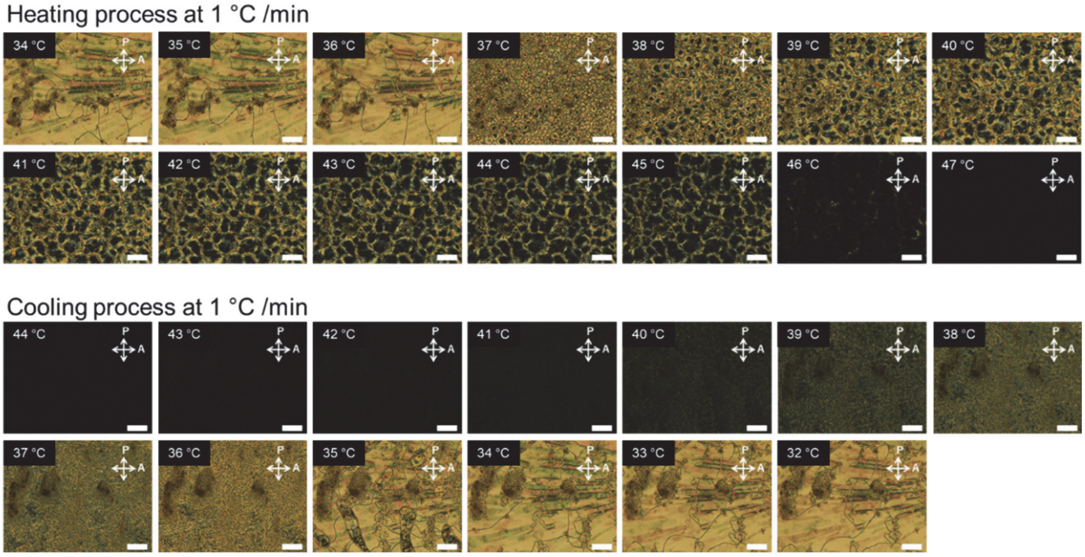

Figure S2. POM images of TR5-doped 5CB containing 10 wt% of LC polymer-grafted nanorods during heating and cooling processes at 1 °C/min. Scale bars: 50 µm.

Text S2. Miscibility of bare ZnO nanorods with TR5-doped 5CB.

Bare ZnO nanorods, instead of LC polymer-grafted ZnO nanorods, were added to TR5-doped 5CB with a weight fraction of 10 wt% to investigate the miscibility. As shown in Figure S3a, the mixture filled in a cell looks opaque. POM observation revealed the separation of aggregated nanorods from 5CB host (Figure S3b). Because of such an inhomogeneous state of 5CB containing bare nanorods, diffraction rings were not formed when irradiated with a laser beam (Figure S3c). In contrast, LC polymer-grafted nanorods are miscible with 5CB. The resulting transparent mixture exhibited diffraction rings, as shown in the main text. This phenomenon can be attributed to the enhanced miscibility with the host LCs resulting from the surface modification with the LC polymer, which facilitates the cooperative orientation of LC polymer-grafted nanorods and host LCs.

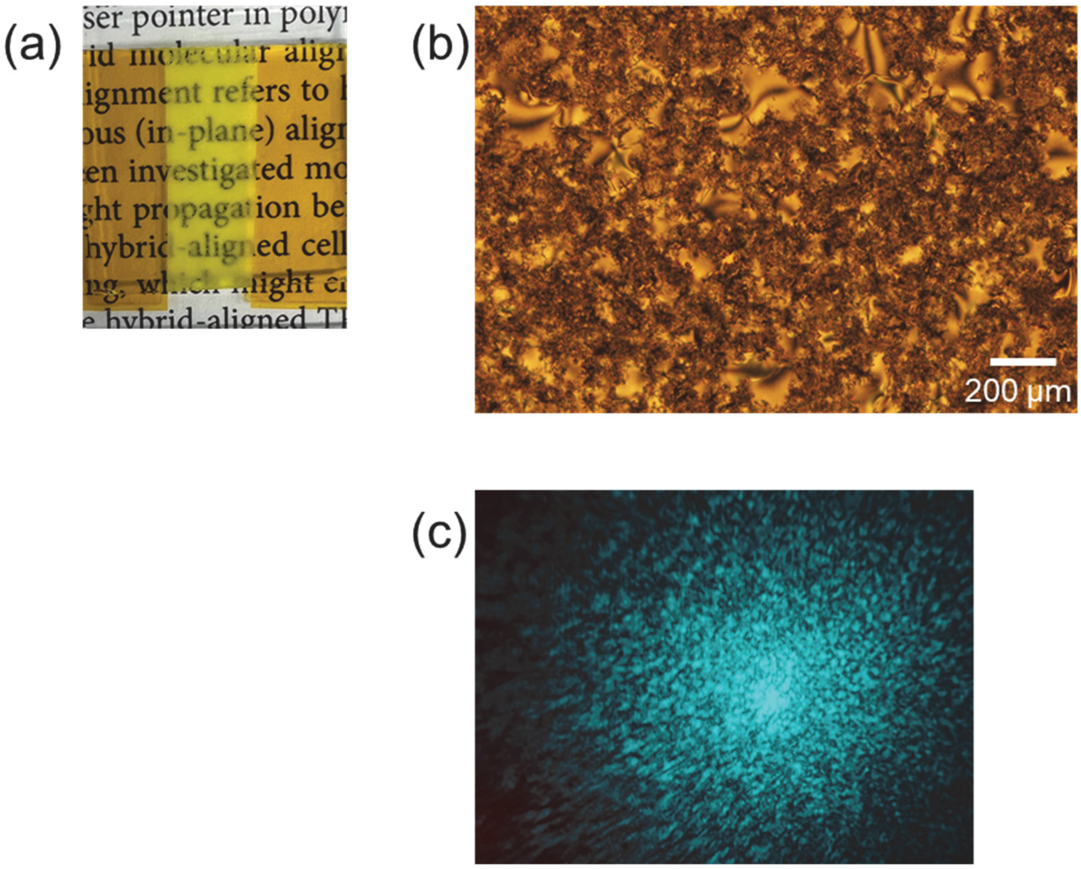

Figure S3. (a) Photograph and (b) POM image of an LC cell of TR5-doped 5CB containing 10 wt% of bare nanorods. (c) Photograph of a transmitted laser beam through the LC cell shown in (a).

Text S3. Optical setup to fabricate and evaluate microlenses

The optical setup used to induce photoinduced molecular reorientation and form microlenses is shown in Figure S4a. A linearly polarized blue laser beam with a wavelength of 488 nm (EXLSR-488C-200-CDRH, Spectra-Physics, MKS Instruments, Inc., Milpitas, California, USA) was incident to the LC cell. The light intensity was controlled using a variable neutral density filter. The incident laser beam power W_0 on the LC cell was calculated by taking the ratio of the beam power split by a non-polarizing cube beam splitter. The light intensity at the irradiation spot was defined as $I = W_0/\pi r^2$, where r is the radius of the laser beam at the focal point of L4, which was controlled to be 50 μm. The laser beam diameter at the focal point was

50 μm. The LC cell was exposed to the laser beam with sufficiently high intensity giving rise to the formation of concentric diffraction ring patterns observed on a white screen placed behind the LC cell. Then, the light intensity was gradually decreased while visually counting the number of rings. Microlenses were obtained by irradiating the sample at high light intensities for a prolonged time. The microlens array was fabricated by irradiating a high-intensity laser beam for 30 min each spot at different locations to form a spatial distribution of microlenses by placing the sample in an *x-y* biaxial stage. A half waveplate (WPQ-4880-2M, Sigma Koki Co. Ltd., Tokyo, Japan) was inserted before L4 to change the polarization direction of the incident laser beam as necessary. The molecular orientation in fabricated microlenses was probed with a 633 nm polarized red laser beam (Melles Griot, 05 LHP 151, Pneum, Saitama, Japan) (Figure S4b). The polarization direction of the probe beam was adjusted to be parallel and perpendicular to the polarization direction that caused the formation of the microlenses. The polarization direction of 5CB in the microlens was further investigated with an orthoscopic POM equipped with a 530 nm retarder plate (U-TP530, Olympus, Tokyo, Japan). The thermal characteristics of the microlens array were investigated with orthoscopic POM equipped with a temperature controller stage (HCS302-MK1000, INSTEC, Inc., Boulder, Colorado, USA).

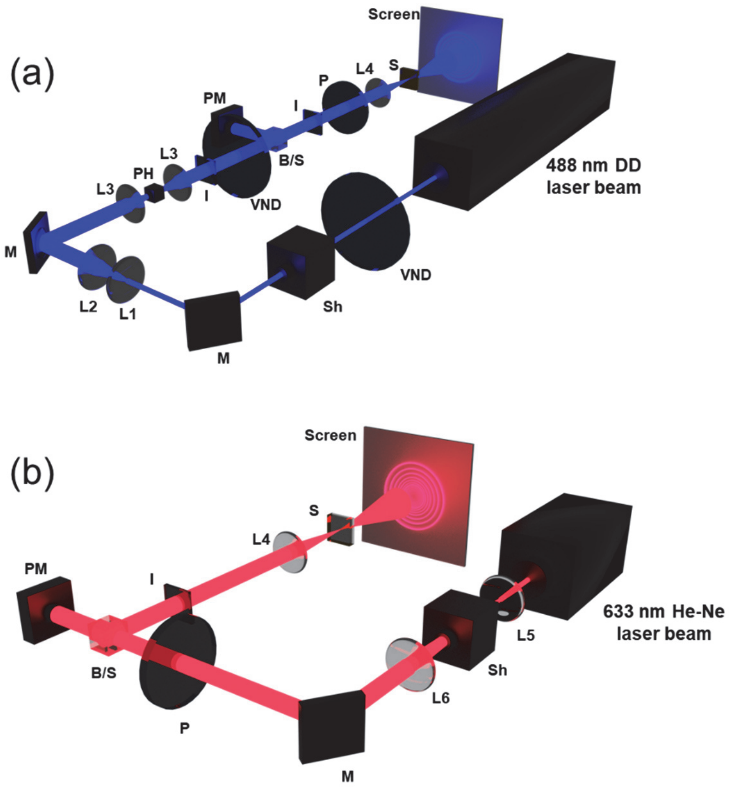

Figure S4. The optical setup used to fabricate (a) and to evaluate (b) the microlens. VND, variable neutral density; Sh, shutter; M, mirror; L1, plane concave lens ($f = -8$ cm); L2, plane convex lens ($f = 20$ cm); L3, plane convex lens ($f = 7.5$ cm); PH, pinhole; I, iris; PM, power meter; B/S, beam splitter; L4, bi-convex lens ($f = 15$ cm); L5, plane concave lens ($f = -12$ cm); L6, plane convex lens ($f = 30$ cm); S, liquid crystal cell.

Text S4. Formation of diffraction rings through TR5-doped 5CB containing homopolymer.

The homopolymer PMA(4OPB) was incorporated into TR5-doped 5CB with the same molar ratio as the polymer grafted on the nanorod, and injected into an LC cell. Diffraction rings were observed by irradiating the sample with the polarized blue laser beam at 71.5 W/cm^2. The diffraction rings ceased when the light intensity was reduced to 1.0 W/cm^2 after the irradiation at 71.5 W/cm^2 for 30 min (Figure S5). The results indicate that TR5-doped 5CB containing the homopolymer without nanorods has no memory effect.

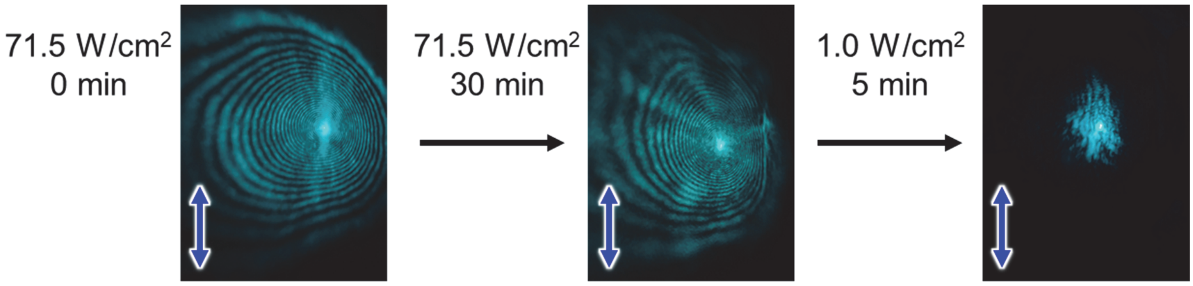

Figure S5. Photographs of the diffraction rings of TR5-doped 5CB containing only the homopolymer PMA(4OPB) with the same polymer concentration as the polymer grafted. The blue arrows represent the polarization direction of the microlenses.

Text S5. Measurement of the focal length of the microlens.

The focal length of the microlens array was experimentally measured under POM by removing the analyzer and sending a linearly polarized light parallel to the polarization direction of the LC molecules in the microlens, as shown in the schematic in Figure S6. The base of the microlens was first focused, and this distance was defined as $Z = 0$ μm. The stage height was then adjusted until a sharp light point was observed on top of the microlens array, and the distance moved was defined as the focal length. This measurement was performed six times to estimate the average focal length.

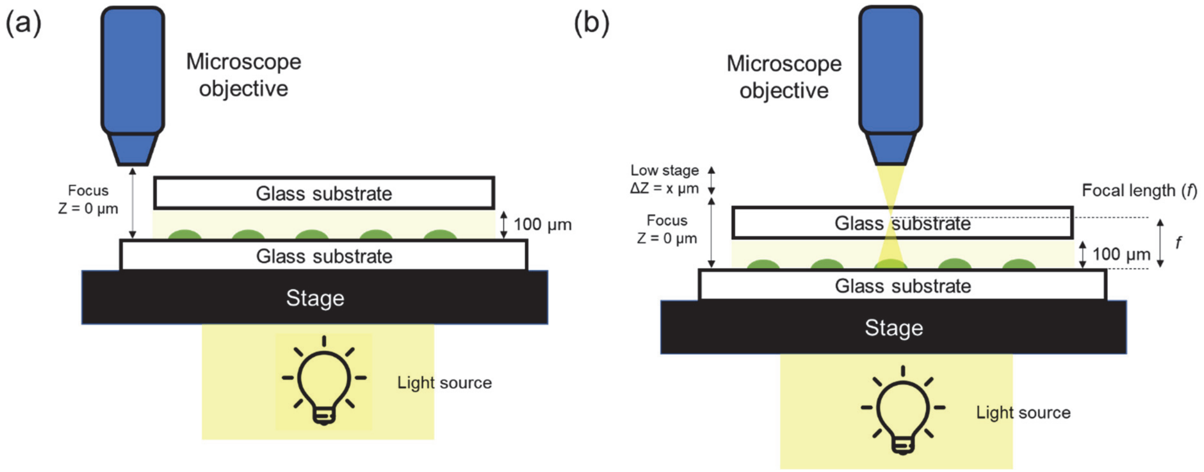

Figure S6. Schematic representation to measure the focal length of the fabricated microlens experimentally. (a) The first step (focus on the microlens) and (b) the second step (focus until the light is focused on the microlens).

Text S6. Long-term stability of microlens arrays.

Microlens arrays were observed by polarized optical microscopy two years after fabrication. Figures S7 and S8 present the POM images at the same place as Figures 10 and 11, respectively. The microlens arrays could be observed with polarization dependence, although some aggregation of the nanorods appeared. The irradiation of the microlens arrays with a blue laser beam (488 nm) at 0.1 W/cm^2 resulted in the formation of diffraction rings (Figure S9). These findings demonstrate the long-term stability of the permanent molecular orientation.

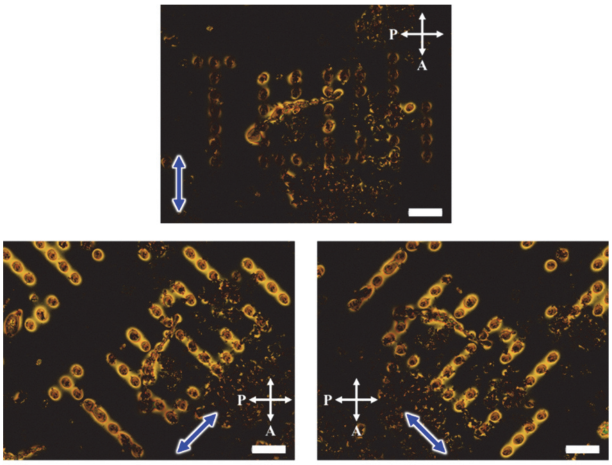

Figure S7. POM images of a microlens array pattern showing the word "Tech." The sample was the same as that shown in Figure 10 in the main text and observed two years after fabrication. The sample was rotated 45° sideways to confirm the polarization direction of the molecules in each microlens. The blue arrow represents the polarization direction of the polarized laser beam used to fabricate each microlens. The scale bar is 200 μm.

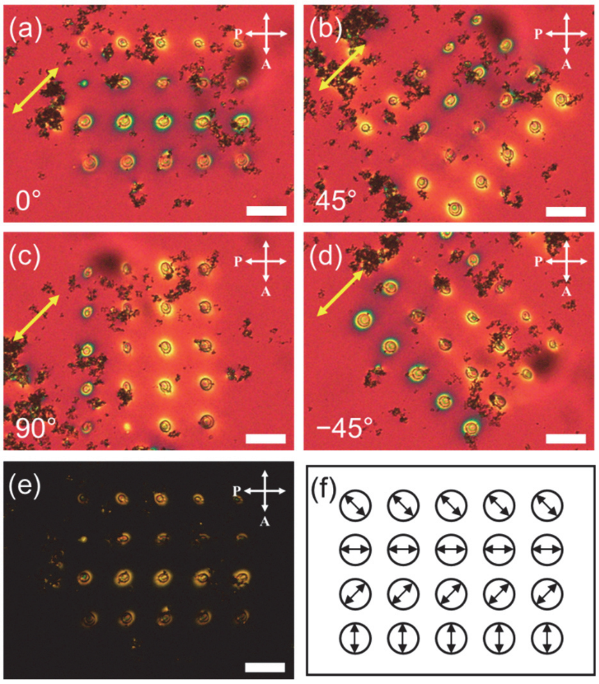

Figure S8. POM images of the microlens array fabricated with different polarization directions. The sample was identical to that shown in Figure 11 in the main text and observed two years after fabrication. The LC cell containing the microlens array was rotated in a counter-clockwise

direction. (a) 0°, (b) 45°, (c) 90°, and (d) −45° equipped with a 530 nm tint plate. (e) POM image of the microlens array with a tint plate. (f) Schematic representation of the molecular alignment in each microlens. The yellow arrow represents the optical axis of the tint plate. The scale bar is 100 μm.

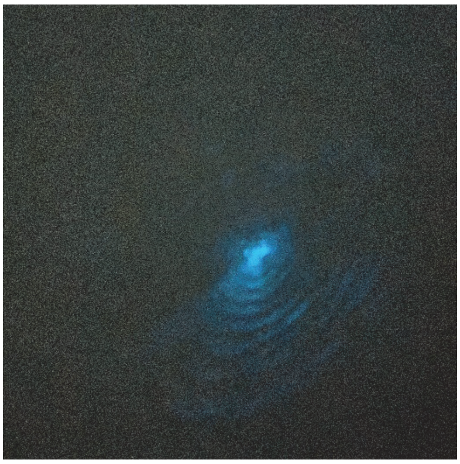

Figure S9. Photograph of diffraction rings through the microlens observed two years after fabrication by the irradiation with a polarized blue laser beam at 0.1 W/cm^2.
